# Incidence of Ovarian Metastases in Reproductive-Aged Women with Appendiceal Cancer: Implications for Fertility Preservation

**DOI:** 10.1245/s10434-026-20315-x

**Published:** 2026-07-30

**Authors:** Morgan Bou Zerdan, Sarah Hmaidan, Hailie Ciomperlik, Joya Ghaleb, Charlotte Sun, Jeffrey A. How, Laura Detti, Terri Woodard, Pamela T. Soliman, Laurie J. McKenzie

**Affiliations:** 1 Department of Gynecologic Oncology and Reproductive Medicine, The University of Texas MD Anderson Cancer Center, Houston, TX USA; 2 Division of Reproductive Endocrinology and Infertility, Department of Obstetrics and Gynecology, Baylor College of Medicine, Houston, TX USA; 3 Faculty of Medicine and Medicinal Sciences, University of Balamand, Beirut, Lebanon

## Abstract

**Purpose:**

Incidence of appendiceal cancer is increasing, with one-third of patients diagnosed before age 50 years. Fertility preservation (FP) is important for patients who have not completed childbearing; however, the safety of oocyte and embryo banking remains unclear due to concerns for ovarian metastases. This study evaluates incidence of ovarian metastases at cytoreductive surgery (CRS) and describes FP practices and outcomes among reproductive-aged women with appendiceal cancer.

**Methods:**

A retrospective review was conducted of female patients aged 18–45 years treated for appendiceal cancer at MD Anderson Cancer Center from January 2020 to January 2025. Clinical, surgical, pathologic, and fertility-related variables were collected. Descriptive statistics assessed rates and laterality of ovarian metastases, FP practices, and reproductive outcomes.

**Results:**

A total of 100 reproductive-aged women (median age 35.9 years, range 19–44 years) with histology-confirmed appendiceal cancer were identified, of whom 47 underwent CRS. Ovarian metastases were documented in 89.4% (42/47), with bilateral involvement in 71.4% (30/42) and unilateral involvement in 28.6% (12/42). Fertility-sparing surgery included retention of one ovary in 25.5% (12/47) and uterine preservation in 19.1% (9/47). Among patients retaining an ovary, 60% developed amenorrhea after adjuvant therapy. Desire for FP was documented in 23.4% (11/47), all were referred for oncofertility consultation. Five patients underwent oocyte or embryo cryopreservation before CRS, including three with confirmed ovarian involvement.

**Conclusion:**

Reproductive-aged women with appendiceal cancer have a high and often bilateral incidence of ovarian metastases, with important implications for FP safety. Individualized fertility counseling prior to CRS is essential to optimize reproductive outcomes while maintaining oncologic safety.

**Supplementary Information:**

The online version contains supplementary material available at https://doi.org/10.1245/s10434-026-20315-x.

Approximately 3000 new cases of appendiceal cancer are diagnosed annually in the USA,^1^ with increasing incidence across all age groups, sexes, and histologic subtypes.^2–5^ For female patients of childbearing age, the incidence of appendiceal adenocarcinoma has increased more than three- to fourfold in recent decades, reflecting a rising disease burden among younger patients.^3^ Cytoreductive surgery (CRS), the standard of care management for appendiceal cancer, often requires bilateral oophorectomy to optimize cytoreduction and reduce recurrence risk in female patients.^2,6^ For nearly one-third of patients diagnosed under age 50 years, this has significant implications for future childbearing.^6^ Guidelines from the American Society for Reproductive Medicine (ASRM) and the American Society of Clinical Oncology (ASCO) recommend early, pretreatment fertility preservation (FP) counseling for patients at risk of treatment-related infertility, however, the safety and feasibility of FP in this population is undetermined.

Ovarian metastases are often identified intraoperatively in patients undergoing CRS for appendiceal cancer, which has implications for patients considering FP.^6^ Bignell et al. reported a large series of 504 female patients who underwent CRS for peritoneal malignancy, of whom 258 had at least one ovary present at time of surgery, with a median age of 56 years (range 19–83 years). Among 352 patients with appendiceal tumors, ovarian metastases were identified in 58.1%, including microscopic involvement in 18.2% of patients whose ovaries appeared grossly normal intraoperatively.^7^ While these findings demonstrate the high prevalence of ovarian involvement in patients with appendiceal cancer, the current data do not specifically address fertility preservation considerations and outcomes among reproductive-aged women.

For reproductive-aged women, the surgical management and pathological considerations of appendiceal cancer introduce unique fertility-related challenges during treatment planning. Specifically, the safety and feasibility of ovarian stimulation and oocyte retrieval in the setting of potential ovarian involvement have not been previously evaluated. For patients contemplating ovarian tissue cryopreservation, a high rate of ovarian metastases would preclude the option of reimplanting ovarian tissue. The primary objective of this study was to determine the incidence of ovarian metastases at the time of CRS among reproductive-aged women and assess oncofertility referral patterns and FP outcomes.

## Patients and Methods

A single-institution retrospective study was conducted at the University of Texas MD Anderson Cancer Center. Eligible patients were women aged 18–45 years with histologically confirmed appendiceal cancer who were evaluated between 1 January 2020 and 1 January 2025. For the primary analysis, we included patients who underwent CRS and had ovarian pathologic assessment available following surgical resection at the time of CRS. Ovarian involvement was defined as pathologically confirmed metastatic appendiceal tumor identified in one or both ovaries on final surgical pathology after cytoreductive surgery, requiring surgical removal of the involved ovary or ovaries (unilateral or bilateral oophorectomy) as part of CRS. Ovarian pathology was not assessed by biopsy. Patients were excluded from the analytic cohort if they had undergone bilateral oophorectomy prior to CRS or if ovarian pathology could not be assessed. Patients who did not undergo CRS were also excluded from the primary analysis, including those with localized disease managed with non-CRS surgical approaches (e.g., right hemicolectomy), low-grade neuroendocrine tumors not requiring CRS, patients with no indication for further cytoreduction following prior surgery, or patients managed with systemic therapy alone.

Clinical, surgical, pathologic, and reproductive-related data were manually abstracted from the electronic medical record. Collected variables included demographics (age, race, ethnicity, state of residence), medical, and oncologic history. Surgical findings (including gross ovarian appearance/involvement), tumor histology, grade, stage, and molecular profile were recorded. Treatment exposures included receipt of hyperthermic intraperitoneal chemotherapy (HIPEC), systemic chemotherapy, both HIPEC and systemic chemotherapy, or neither.

Reproductive data included gravidity and parity history, menstrual history, documented interest in FP, and receipt of oncofertility referral and consultation. Desire for FP and oncofertility referral patterns were assessed through retrospective chart review of preoperative documentation, including clinical notes from the treating surgeon, oncology team, and consulting services. Fertility-sparing surgical management was defined as preservation of the uterus and/or at least one ovary at the time of cytoreductive surgery, and details regarding ovarian (unilateral or bilateral) and/or uterine preservation were recorded. Fertility preservation procedures were categorized as oocyte, embryo, or ovarian tissue cryopreservation, and the number of gametes obtained was documented. Pregnancy attempts and pregnancy outcomes were abstracted when available.

Descriptive statistics (frequencies, percentages, medians, means, and ranges) were used to summarize demographics, oncologic, surgical, and fertility-related variables. Continuous variables were summarized using measures of central tendency and dispersion, while categorical variables were reported as counts and proportions. Crosstabulations were used to descriptively compare ovarian involvement across selected clinicopathology features, such as histologic subtype. Overall survival was estimated using life table analysis, a nonparametric method for time-to-event data that accounts for censoring and varying follow-up times. Survival probabilities were reported at clinically relevant timepoints. Due to the limited sample size and heterogeneity in treatment approaches, no multivariable modeling or survival analyses were conducted. All analyses were performed using IBM SPSS (version 29.0.1.0). All analyses complied with institutional ethical and confidentiality standards, and results were reported in aggregate to protect patient privacy. Institutional review board (IRB) approval was obtained at MD Anderson (IRB# 2025-0685).

## Results

### Demographics

Among 100 women aged 18–45 years with histologically confirmed appendiceal cancer identified during the study period, 47 underwent CRS and comprised the analytic cohort. The remaining 53 patients did not undergo CRS and were excluded on the basis of the prespecified cohort definition. Reasons cited for avoidance of CRS were negative margins at initial surgery (28.3%, 15/53), low-grade neuroendocrine tumors not requiring CRS (32.1%, 17/53), treatment with right hemicolectomy per National Comprehensive Cancer Network (NCCN) guideline-based recommendations for appendiceal adenocarcinoma and select neuroendocrine tumors (24.5%, 13/53), and localized disease or chemotherapy alone without CRS (15.1%, 8/53). Among the 47 reproductive-aged women undergoing CRS for appendiceal cancer, the mean age was 36.4 years (range 19–43 years). Most patients were white non-Hispanic (92.5%, 43/47) and 36.2% (17/47) were nulligravid at diagnosis. Staging was as follows: 2.2% (1/47) stage II, 4.3% (2/47) stage III, and 93.6% (44/47) stage IV. The most common histological subtypes included mucinous adenocarcinoma (34.0%, 16/47), low-grade appendiceal mucinous neoplasm (LAMN) (3.9%, 15/47), and goblet cell adenocarcinoma (10.6%, 5/47) (Supplementary Fig. 1). Tumor grade was documented in 42/47 of patients with a distribution of 34.6% grade 1 (16/47), 27.7% grade 2 (13/47), and 27.7% grade 3 (13/47).

**Fig. 1:**
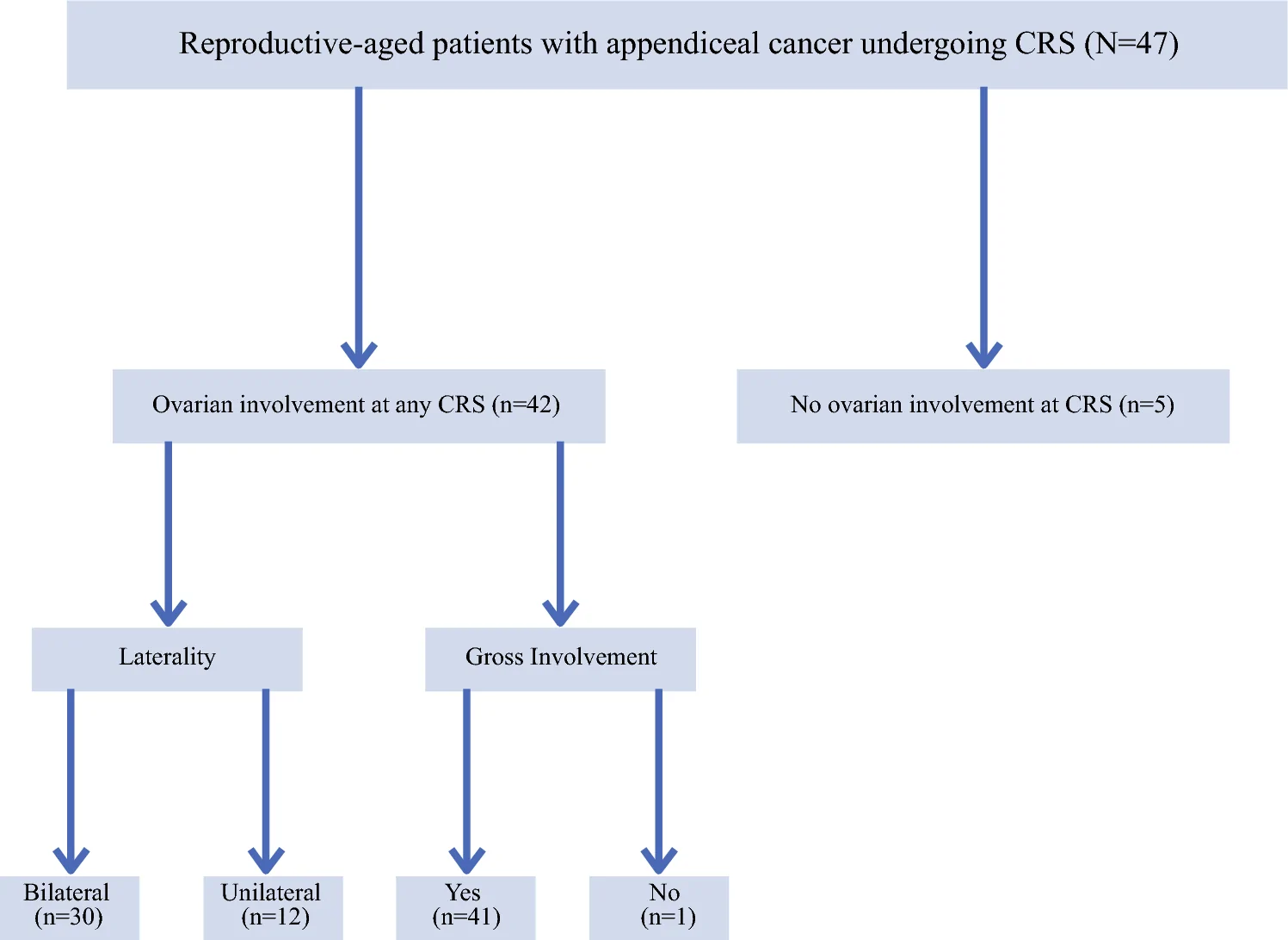
Ovarian involvement at time of cytoreductive surgery (CRS) among reproductive aged women with appendiceal cancer

**Fig. 2:**
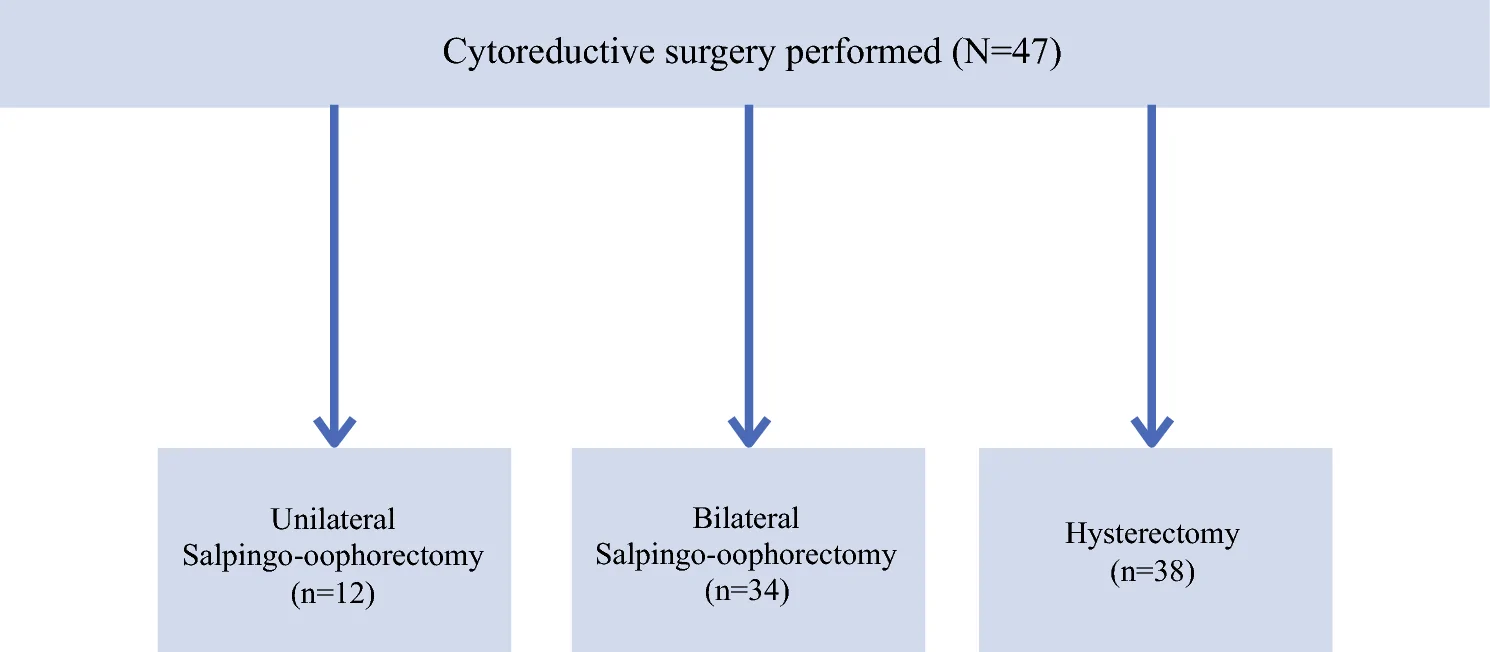
Type of fertility impacting surgery performed at time of CRS among reproductive aged women with appendiceal cancer (*n* = 47)

Ovarian involvement was pathologically confirmed in 89.3% (42/47) of patients, of whom the vast majority had grossly visible ovarian disease documented at time of surgery (41/42) (Fig. 1). Among the 42 women with ovarian metastases, 71.4% (30/42) had bilateral disease and 28.6% (12/42) had unilateral involvement (Fig. 1). Bilateral salpingo-oophorectomy was performed in 72.3% (34/47) of patients, unilateral salpingo-oophorectomy in 25.5% (12/47), and concurrent hysterectomy in 80.9% (38/47) (Fig. 2). One patient (2.1%, 1/47) underwent hysterectomy without oophorectomy.

Ovarian involvement did not differ significantly on the basis of histologic type and was documented in 73.3% (11/15) of LAMN, 100% (4/4) of high-grade appendiceal mucinous neoplasm (HAMN), and 100% (16/16) of mucinous adenocarcinomas, (5/5) goblet cell adenocarcinomas, (3/3) signet ring cell carcinomas, (2/2) adenocarcinomas (NOS), and (1/1) poorly differentiated neuroendocrine carcinoma. High rates of ovarian metastases were observed across all tumor grades, including 100% (13/13) of grade 3 tumors, 92.3% (12/13) of grade 2 tumors, and 93.8% (15/16) of grade 1 tumors (Supplementary Fig. 2). Ovarian involvement at time of CRS was documented in 2.4% (1/42) of patients with stage III disease and 97.6% of patients with stage IV (41/42). The single patient classified as stage III was staged on the basis of findings at initial diagnosis; ovarian metastasis was not identified until 3 years later at time of CRS.^8^

Hyperthermic intraperitoneal chemotherapy (HIPEC) alone was performed in 40.4% (19/47) of patients, HIPEC plus chemotherapy in 34% (16/47), chemotherapy alone in 14.8% (7/47), and no HIPEC/chemotherapy in 10.6% (5/47). Among the 12 women who retained at least one ovary, 9 underwent HIPEC, systemic chemotherapy, or both, while 3 did not receive these treatments. All three patients who did not receive HIPEC or chemotherapy maintained normal menstrual cycles. In contrast, menstrual abnormalities were observed among those who received treatment. Following HIPEC alone, 66.7% (2/3) developed amenorrhea and 33.3% (1/3) had irregular cycles. After chemotherapy alone, amenorrhea and irregular menses each occurred in 50.0% (2/4). After combined HIPEC plus chemotherapy, 50% (1/2) developed amenorrhea and 50% (1/2) had irregular menses.

Desire for FP was documented in 23.4% (11/47) of patients, and all were referred for oncofertility consultation. In addition, 9 patients (19.1%, 9/47) declined oncofertility consultation, and 27 patients (57.4%, 27/47) had no documentation of FP counseling or oncofertility referral within their medical record. Five patients completed FP and their staging was as follows: 20% (1/5) stage 2, 20% (1/5) stage 3, and 60% (3/5) stage 4. Two patients underwent oocyte cryopreservation, and three underwent embryo cryopreservation. Histologic subtypes among patients undergoing FP included one LAMN, one signet ring cell carcinoma, one HAMN, one non-mucinous adenocarcinoma, and one goblet cell adenocarcinoma. The median age of patients who pursued FP was 32.6 years (range 25–38 years).

Among patients who underwent FP, the mean number of oocytes retrieved was 16 (range 12–23), with a mean of 13 oocytes cryopreserved (range 9–17). Three patients underwent embryo cryopreservation with a mean of 5 embryos cryopreserved (range 4–7). No surgical complications were reported at time of transvaginal oocyte harvest.

Baseline ovarian reserve and additional stimulation data were available for two of the five patients who underwent FP. Anti-Müllerian hormone levels ranged from 3.2 to 3.9 ng/mL. Duration of ovarian stimulation ranged from 10 to 12 days, with peak estradiol levels ranging from 2842 to 4199 pg/mL. Oocyte maturity rate ranged from 25 to 73.9%.

Among the five patients who completed FP, none demonstrated evidence of ovarian involvement per preoperative CT imaging. Three patients subsequently underwent bilateral salpingo-oophorectomy with hysterectomy, one patient underwent unilateral salpingo-oopherctomy with hysterectomy, and one did not undergo fertility-limiting surgery at time of CRS, with ovaries and uterus left in situ. Gross ovarian involvement was reported in all patients who underwent definitive surgery; however, pathologically confirmed ovarian metastases were found in 75% (3/4) of patients. In the remaining patient, ovarian pathology demonstrated fibrous tissue with acellular mucin and calcifications without evidence of malignancy.

Sixteen months following surgical treatment, one patient in the oocyte cryopreservation group who underwent fertility-sparing surgery conceived spontaneously and delivered a healthy-term singleton without reported pregnancy-related complications. One patient in the embryo cryopreservation group transferred an embryo to a gestational carrier, which did not result in a pregnancy. At the time of manuscript preparation, no patients in the oocyte cryopreservation group had utilized their cryopreserved oocytes.

Six patients who underwent oncofertility consultation did not proceed with FP. Of these, two elected fertility-sparing surgical management, two deferred FP due to safety concerns, one declined due to timing constraints, and FP decision-making was unknown for the remaining patient who pursued consultation with a local reproductive endocrinologist outside the institution.

At time of last follow-up, 61.7% (29/47) of patients were alive with no evidence of disease, 31.9% (15/47) were alive with disease, and 6.4% (3/47) were deceased. The median follow-up time for the cohort was 21.7 months (range 6.8–80.0 months). Overall survival was estimated using life table analysis. The 3-year overall survival rate was 97%, and the 5-year overall survival rate was 77%. Of the patients who underwent FP, 60% (3/5) were alive with no evidence of disease and 40% (2/5) were alive with disease. Due to the limited number of events and heterogeneity in treatment approaches, no further survival modeling was performed.

## Discussion

We found a high (89.4%) incidence of ovarian metastases among reproductive-aged women with appendiceal cancer undergoing CRS, with most patients demonstrating bilateral ovarian disease. This has significant implications for reproductive decision-making, including safety of ovarian stimulation and oocyte harvest. Ovarian stimulation requires approximately 8–12 days of exogenous gonadotropins to mature the oocytes followed by oocyte aspiration. The aspiration (egg harvest) is facilitated vaginally with a 16-gauge 35 cm in length aspiration needle, and multiple ovarian punctures are required. Concerns for oocyte harvest include ovarian tumor involvement, which may increase risk of bleeding as well as potential for tumor spread. Ovarian tissue cryopreservation (OTC) is another fertility preservation strategy that involves harvesting and cryopreserving cortical ovarian tissue. When fertility restoration is desired, the cryopreserved tissue is thawed and autotransplanted to restore reproductive potential. However, on the basis of our findings, reimplantation might not be an option in this patient population, due to concerns for tumor reseeding when microscopic metastases are present.

Desire for FP was documented in approximately one-quarter of patients, and all patients with documented interest received an oncofertility consultation. However, the majority of patients did not have documented FP counseling in the medical record, limiting the ability to determine whether reproductive goals were assessed or discussed. Despite this, only a small subset of patients completed oocyte or embryo cryopreservation prior to CRS (5/47). Among patients who did not proceed with FP after consultation, reasons included pursuit of fertility-sparing surgical management, safety concerns related to oocyte harvest, and timing constraints, highlighting the complex decision-making that occurs in this population. For the small group of women who underwent oocyte harvest, no short-term adverse outcomes were found. Notably, the histologic subtypes among patients who underwent FP spanned a clinically diverse spectrum, including LAMN, HAMN, goblet cell adenocarcinoma, signet ring carcinoma, and non-mucinous adenocarcinoma. This heterogeneity is significant, as more indolent histology may afford greater time for FP, whereas aggressive subtypes may necessitate expedited surgical intervention, hastening or precluding the window for FP.

For appendiceal and other lower gastrointestinal peritoneal malignancies, fertility-sparing surgery is frequently unavoidable due to the need for complete cytoreduction.^9^ Existing literature on fertility outcomes following CRS largely addresses mixed peritoneal malignancy cohorts treated with CRS-HIPEC, and appendiceal-specific data in reproductive-aged women remain limited. In these broader CRS-HIPEC cohorts, few patients retain childbearing potential due to the high rate of hysterectomy and oophorectomy at the time of surgery, and fertility counseling prior to surgery remains inconsistent and underutilized.^9,10^ Childbearing after CRS-HIPEC has been reported in women who conserve their genital organs, reinforcing the importance of preoperative fertility discussions.^11^ To our knowledge, this study is the first to characterize ovarian metastasis rates, oncofertility referral patterns, and FP outcomes specifically in reproductive-aged women with appendiceal cancer undergoing CRS, addressing a meaningful gap in the oncofertility literature. Referral prior to CRS planning allows for individualized risk–benefit assessment that accounts for disease burden, surgical urgency, and potential safety concerns related to ovarian stimulation and egg harvest.^12^ Documentation of these discussions helps ensure that fertility-related values and preferences are consistently elicited and incorporated into treatment planning. Apart from facilitating FP, oncofertility counseling plays an important psychosocial role by supporting patients who may otherwise face loss of genetic parenthood and its associated long-term emotional and social consequences.^13^ These discussions may include third-party reproductive options such as donor gametes, gestational carriers, or adoption, reinforcing patient autonomy in the setting of fertility-limiting oncologic care.

To our knowledge, this is the first study evaluating the rate of appendiceal-associated ovarian metastases in the reproductive-aged population. Strengths of this study include its focus on a defined cohort of reproductive-aged patients and the availability of detailed surgical and pathologic data from a high-volume NCI cancer center. Limitations include the retrospective study design, the modest number of patients who underwent fertility-sparing CRS, number of patients who completed FP, and the predominantly white, non-Hispanic composition of the study population (92.5%). Additionally, Peritoneal Cancer Index (PCI) data were not collected as part of this retrospective study and therefore could not be analyzed. As a high-volume tertiary referral center, MD Anderson Cancer Center may disproportionately manage patients with advanced and complex disease, which may have contributed to the particularly high incidence of ovarian metastases observed in this cohort and may limit generalizability to other CRS-HIPEC centers treating appendiceal cancer. For patients who underwent oocyte harvest despite the presence of ovarian metastases, the limited duration of follow-up and sample size preclude definitive conclusions regarding oncologic outcomes, including recurrence risk.

Among the patients who completed FP, preoperative CT imaging did not reliably identify ovarian involvement, as ovaries without radiographic evidence of disease demonstrated gross ovarian involvement in all cases and pathologically confirmed ovarian metastases in 75% (3/4) of patients at time of CRS. Fortunately, despite documentation of gross and pathologic ovarian involvement at time of CRS, no bleeding complications were reported during oocyte harvest in this small cohort.

Future efforts should focus on multidisciplinary collaboration, standardized pre- and posttreatment assessment of ovarian function for those undergoing fertility-sparing CRS, and clear pathways for early oncofertility referral. Recent guidance from ASCO also highlights the importance of incorporating ovarian toxicity monitoring, using both clinical measures and biomarkers, to better understand treatment-related effects on ovarian function over time.^14^ Incorporating structured counseling and documentation tools may also improve the consistency and visibility of fertility-related discussions across multidisciplinary care teams. Considering the high incidence of ovarian metastases observed in this study, ovarian tissue cryopreservation as a FP strategy in this population should be approached with caution due to the theoretical risk of tumor reseeding at the time of reimplantation. Long-term oncologic follow-up is also needed to determine the safety of both ovarian tissue cryopreservation and oocyte aspiration related to tumor spread. Additionally, the increasing nonoperative management of appendicitis may delay diagnosis of occult appendiceal malignancies in younger patients, potentially increasing the risk of advanced disease and need for fertility-limiting treatment.^14^ Furthermore, psychosocial counseling referrals and utilization of supportive care services were not systematically captured in this study. Future work should examine the incidence and impact of psychosocial support and care navigation referrals in this population, as these represent important components of comprehensive oncofertility care for reproductive-aged women facing fertility-limiting cancer treatment. Given the high incidence of ovarian metastases and low utilization of FP, individualized counseling is essential, and prospective studies are needed to define the safety, feasibility, and long-term outcomes of FP strategies in this understudied population.

## Conclusions

In this cohort of reproductive-aged women with appendiceal cancer, ovarian metastases were common and often bilateral, underscoring the substantial reproductive impact of standard surgical management. We found that FP was underutilized, which may reflect the challenge of integrating fertility goals with oncologic urgency, but also may reflect the uncertainty of safe, fertility-preserving options for these young women with cancer. Early referral for oncofertility counseling may better allow for individualized risk assessment, clarify procedural uncertainties, and align reproductive goals with oncologic priorities. These findings highlight the need for disease-specific, multidisciplinary oncofertility frameworks for appendiceal cancer that explicitly address the high prevalence of ovarian metastases and the unresolved safety considerations surrounding FP.

## Supplementary Information

Below is the link to the electronic supplementary material.

Supplementary file1 (DOCX 238 KB)
