# Supplementary material for: Incidence of Ovarian Metastases in Reproductive-Aged Women with Appendiceal Cancer: Implications for Fertility Preservation
Source: Ann Surg Oncol. 2026 Jul 30;33(12):10205–11. doi: 10.1245/s10434-026-20315-x (PMC13675260; doi:10.1245/s10434-026-20315-x)
Supplement: Supplementary file 1 [file 10434_2026_20315_MOESM1_ESM.docx]

**Supplementary Figures:**

Supplementary Figure 1: pathology confirmed ovarian metastasis at the time of CRS among reproductive aged women with appendiceal cancer: Stratified by tumor histology (n=47)

Supplementary Figure 2: Pathology confirmed ovarian metastasis at the time of CRS among reproductive aged women with appendiceal cancer: Stratified by tumor stage (n=47)
